# Time-Restricted Eating Versus Daily Calorie Restriction: Effect on Sleep in Adults with Obesity over 12 Months

**DOI:** 10.3390/nu16203528

**Published:** 2024-10-18

**Authors:** Shuhao Lin, Sofia Cienfuegos, Mark Ezpeleta, Kelsey Gabel, Vasiliki Pavlou, Shaina J. Alexandria, Krista A. Varady

**Affiliations:** 1 Department of Kinesiology and Nutrition, University of Illinois Chicago, Chicago, IL 60607, USA; slin89@uic.edu (S.L.); scienf2@uic.edu (S.C.); kdipma2@uic.edu (K.G.); pavlou2@uic.edu (V.P.); 2 Division of Endocrinology, Metabolism and Diabetes, University of Colorado School of Medicine, Aurora, CO 80045, USA; mezpel2@uic.edu; 3 Department of Preventative Medicine (Biostatistics), Northwestern University, Chicago, IL 60208, USA; shaina.alexandria@northwestern.edu

**Keywords:** time-restricted eating, intermittent fasting, calorie restriction, sleep quality, insomnia, obstructive sleep apnea, weight loss, obesity

## Abstract

**Objective:** The aim of this secondary analysis was to compare the effects of time-restricted eating (TRE) versus daily calorie restriction (CR) on sleep quality, duration, insomnia severity, and risk of obstructive sleep apnea in adults with obesity over one year. **Methods:** A total of 90 participants were randomized to one of three groups for 12 months: 8 h TRE (eating only between 12 p.m. and 8 p.m.); CR (25% daily calorie restriction) or a no-intervention control group. **Results:** By the end of the study, weight loss was 4.61 kg (95% CI; 7.37 to 1.85 kg; *p* ≤ 0.01) for the TRE group and 5.42 kg (CI; 9.13 to 1.71 kg; *p* ≤ 0.01) for the CR group, with no statistically significant difference between TRE and CR (0.81 kg [CI; 3.07 to 4.69]; *p* = 0.68]). Self-reported sleep quality, sleep duration, insomnia severity, and risk of obstructive sleep apnea did not change in the TRE or CR groups versus controls by month 12. **Conclusions:** These findings suggest that the weight loss produced by TRE and CR does not have any impact on various sleep parameters in adults with obesity over one year.

## 1. Introduction

Obesity and sleep quality have an inverse and bidirectional relationship [1]. Studies show that short sleep duration and poor sleep quality can increase energy intake [2,3], decrease diet quality [2], and increase the risk for developing obesity [4,5]. Additionally, elevated body mass index (BMI > 25 kg/m^2^) has been associated with decreased sleep duration, sleep quality, and increased risk of obstructive sleep apnea [6]. It has also been shown that weight loss may be helpful in improving sleep quality and decreasing the risk of obstructive sleep apnea in patients with obesity [7,8].

Time-restricted eating (TRE) has gained popularity as an alternative weight loss strategy, when compared to traditional calorie restriction (CR). TRE involves confining the eating window to 4 to 10 h per day and fasting with energy-free beverages for the remaining hours of the day. Previous studies have shown that TRE results in mild to moderate weight loss (3–5% over 2–12 months) and improvements in metabolic health parameters such as blood pressure and insulin sensitivity [9]. Furthermore, it has been proposed that TRE may help individuals align their eating schedules to their circadian rhythms, leading to improvements in body weight [10] and sleep [11]. To date, only a handful of studies have looked at the effect of TRE on sleep [12]. Although most studies found that TRE has no effect on sleep outcomes, these trials had small sample sizes, were short in duration (1–3 months), and many lacked control groups. Moreover, no study to date has directly compared the effects of TRE versus a standard care diet (daily calorie restriction, CR) on sleep outcomes.

We recently conducted a 12-month study to compare the effect of TRE versus CR on body weight and metabolic health endpoints [13]. We found that TRE and CR produced similar weight loss (approximately 5%) in adults with obesity, compared to controls, after 12 months. However, our original study [14] did not assess how these two diet protocols influenced sleep. Given the increasing popularity of the TRE diet, it is crucial to understand how it may affect sleep compared to traditional dieting (CR). These data may help further inform clinical decision making in the dietary weight loss field.

Accordingly, the aim of this secondary analysis was to compare the effects of TRE versus CR on sleep quality, duration, insomnia severity, and risk of obstructive sleep apnea in adults with obesity, over one year. We hypothesized that the TRE and CR interventions would produce similar improvements in sleep quality and duration by month 12, versus controls.

## 2. Methods

### 2.1. Subject Selection

This is a secondary analysis of a 12-month randomized controlled trial [14]. The Office for the Protection of Research Participants at the University of Illinois Chicago approved the experimental protocol (Protocol 2020-1512), and all volunteers gave written informed consent to participate in the trial. Participants were recruited from the Chicagoland area through posters placed around the University of Illinois at Chicago campus and surrounding area. Inclusion criteria were as follows: age between 18 and 65 years, and BMI between 30 and 50 kg/m^2^. This BMI range was chosen as these individuals are ideal candidates for dietary weight loss interventions, as stipulated by the current obesity management guidelines [15]. Exclusion criteria were as follows: history of diabetes mellitus, use of weight loss medications, weight unstable for 3 months before the beginning of the study (>4 kg weight loss or gain), eating within less than a 10 h window, perimenopausal or otherwise irregular menstrual cycle, nightshift worker, pregnant or trying to become pregnant, and current smokers. Eligible participants were randomized through a stratified randomization procedure (based on sex, age, and BMI) into three groups: TRE, CR, or control, as previously described [14]. The 12-month trial was divided into a 6-month weight loss phase followed by a 6-month weight maintenance phase.

### 2.2. Time-Restricted Eating Group Protocol

During the 6-month weight loss phase, participants in the TRE group were instructed to eat ad libitum between 12:00 and 8:00 p.m. daily (8 h eating window) and to fast from 8:00 to 12:00 p.m. the following day (16 h fasting window). Participants did not have to monitor food or energy intake during the 8 h eating window. During the 16 h fasting window, participants were allowed to drink water and energy-free beverages like black coffee, tea, and diet sodas (limit 2 per day). During the 6-month weight maintenance period, participants were asked to maintain their weight by expanding their eating window to 10:00 a.m. to 8:00 p.m. (10 h eating window) and fast from 8:00 p.m. to 10:00 a.m. (14 h fasting window). TRE participants received diet counseling (over Zoom) weekly during the first 3 months and then biweekly from months 4 to 12 to learn how to make healthy eating choices [16].

### 2.3. Calorie Restriction Group Protocol

During the 6-month weight loss phase, participants in the CR group were instructed to reduce their energy intake by 25% each day. Total energy expenditure was calculated using the Mifflin equation [17] and multiplied by the appropriate activity factor for each participant. CR participants met with the study dietitian at the beginning of the study to develop weight loss meal plans according to their food preferences. During the 6-month weight maintenance phase, CR participants were instructed to consume 100% of their newly calculated energy needs. CR participants received diet counseling weekly (over Zoom) during the first 3 months and then biweekly from months 4 to 12 to learn how to make healthy eating choices [16].

### 2.4. Control Group Protocol

Participants in the control group were asked to maintain their weight and not to change their eating and activity habits during the 12-month trial. Controls did not receive diet counseling but were contacted by the study coordinators at the same frequency as the TRE and CR groups to provide body weight measurements.

### 2.5. Outcome Measures

All outcomes were assessed at baseline, month 6, and month 12. Body weight was measured without shoes, in light clothing, using a digital scale at the research center. Body composition (fat mass, lean mass, and visceral fat mass) was measured using dual X-ray absorptiometry (iDXA, GE). Height was measured using a wall-mounted stadiometer. BMI was calculated as kg/m^2^. Energy intake was assessed through a 7-day food record using the Automated Self-Administered 24 h (ASA-24) diet assessment tool [18] at baseline and month 12. Physical activity was measured using a pedometer (Fitbit) worn continuously for 7 days at baseline and month 12.

Chronotype was measured by a 19-item Morningness–Eveningness Questionnaire (MEQ) [19]. Chronotype is defined as an individual’s natural inclination towards the times of day when they prefer to sleep or when they are most alert or energetic. The MEQ gives a sum score range from 16 to 86, which can be categorized into: definitely morning type (70–86), moderately morning type (59–69), intermediate type (42–58), moderately evening type (31–41), and definitely evening type (16–30). Risk of obstructive sleep apnea (% occurrences) was estimated using the 10-item self-report Berlin Questionnaire [20]. Self-reported sleep quality, duration, and timing were measured using the Pittsburgh Sleep Quality Index (PSQI) [21]. The PSQI is a 19-item survey that measures sleep quality in the past month, resulting in a score of 0–21. A score of 5 or higher indicates poor sleep quality. Sleep onset latency was also measured by PSQI. Sleep onset latency is defined as the amount of time it takes a person to fall asleep. The sleep latency score ranges from 0 to 3, with 0 indicating no problem falling asleep and 3 indicating a severe problem. The Insomnia Severity Index (ISI) was used to measure insomnia severity [22]. The ISI is a 7-item self-reported survey that rates each item on a 5-point Likert scale, yielding a total score of 0–28. An ISI score of 0–7 indicates no clinically significant insomnia; a score of 8–14 indicates subthreshold insomnia; a score of 15–21 indicates moderate-severity insomnia; and a score of 22–28 indicates severe insomnia.

### 2.6. Statistical Analyses

Data are shown as mean (95% confidence interval [CI]) unless otherwise noted. We conducted an intention-to-treat analysis, which included data from all 90 participants who underwent randomization. A linear mixed model was used to assess time, group, and time*group effects for each outcome. In each model, time and group effects (and their interaction) were estimated without imposing a linear time trend. Models for body weight (primary study outcome) and body weight percentage included data from 13 time points (baseline and 12 months of follow-up), and models for all other outcomes included data from 3 time points (baseline, month 6, and month 12). To account for multiple comparisons for the primary outcome analysis (TRE-CR, TRE-CON, CR-CON), pairwise group comparisons of absolute body weight were assessed relative to a Bonferroni-adjusted two-tailed *p* value of 0.017. *p* values generated from analyses of secondary outcomes were not adjusted for multiplicity and are considered descriptive. Therefore, *p* values for all other outcomes are compared to the standard 0.05 threshold for significance. Pearson correlations were performed to assess the relationships between changes in body weight, body composition, and sleep measures. All analyses were performed using R software (version 4.3.3).

## 3. Results

### 3.1. Baseline Characteristics and Dropouts

As previously reported [14], a total of 126 participants were screened, and 90 were randomized to the TRE group (*n* = 30), CR group (*n* = 30), or control group (*n* = 30). By the end of the 12-month trial, the number of completers was *n* = 77 (TRE: *n* = 26; CR: *n* = 25; control: *n* = 26). Baseline characteristics of the participants were comparable between groups (Table 1). Participants in the TRE, CR, and control groups were classified in the intermediate chronotype (i.e., MEQ score between 42 and 58).

### 3.2. Body Weight and Body Composition

**Figure 1:**
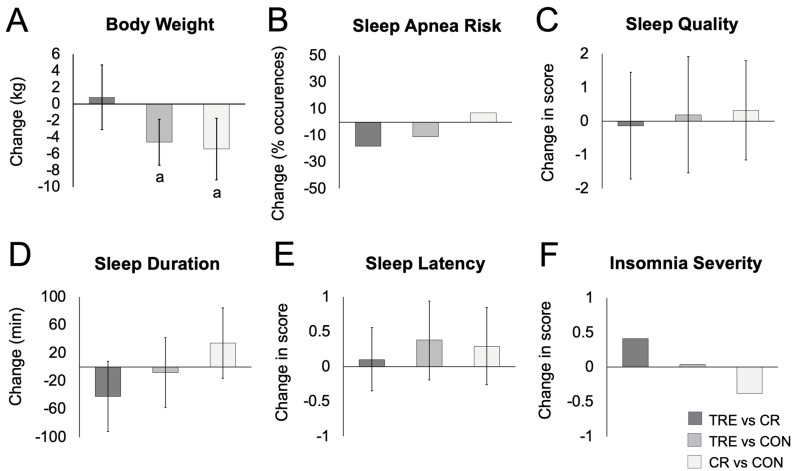
Change in body weight and sleep parameters between TRE, CR, and control groups by month 12. Abbreviations: CON: control group, CR: calorie restriction group, TRE: time-restricted eating group. (**A**) Change in body weight between groups. (**B**) Change in sleep apnea risk between groups. (**C**) Change in sleep quality score between groups (measured by Pittsburgh Sleep Quality Index (PSQI)). (**D**) Change in sleep duration between groups. (**E**) Change in sleep latency score between groups. (**F**) Change in insomnia severity score between groups (measured by the Insomnia Severity Index (ISI)). Means were estimated using an intention-to-treat analysis using a linear mixed model with 95% CIs for each parameter from baseline by diet group. ^a^ Indicates statistical significance using Bonferroni-adjusted 2-tailed *p* < 0.017. All other comparisons were not significant.

Changes in body weight and body composition are reported in Table 2 and Figure 1. By month 12, weight loss was 4.61 kg (95% CI, 7.37 to 1.85 kg; *p* ≤ 0.01) for the TRE group and 5.42 kg (CI, 9.13 to 1.71 kg; *p* ≤ 0.01) for the CR group, with no statistically significant difference between TRE and CR (0.81 kg [CI, 3.07 to 4.69]; *p* = 0.68]). Results for changes in body weight percentage from baseline were similar. By the end of the study, TRE and CR participants experienced reductions in fat mass, waist circumference, and BMI, compared with controls. However, lean mass and visceral fat mass did not change significantly between groups by month 12.

### 3.3. Energy Intake and Physical Activity

As reported previously [14], energy intake (mean ± SD) decreased in both the TRE group (−425 ± 531 kcal/d) and CR group (−405 ± 712 kcal/d) at month 12, with no difference between the TRE and CR groups. Physical activity (steps/day) did not change over time or between groups, as previously described [14].

### 3.4. Sleep Measures

Changes in sleep measures over 12 months are reported in Table 2 and Figure 1. At baseline, the risk for obstructive sleep apnea was present in 54% of TRE participants, 38% of CR participants, and 26% of control participants. Risk of obstructive sleep apnea did not change significantly between groups by month 6 or 12.

At baseline, sleep quality was poor (PSQI scores >5) in the TRE (7.8 ± 3.8), CR (6.8 ± 3.3), and control groups (8.1 ± 2.4). The PSQI sleep quality score did not change significantly between groups by month 6 or month 12. At baseline, the sleep onset latency score was 1.3 ± 1.0 in the TRE group, 1.0 ± 1.0 in the CR group, and 1.6 ± 0.7 in the control group, indicating mild issues with falling asleep in all groups. The sleep onset latency score did not change significantly between groups by month 6 or 12.

At baseline, mean (SD) sleep duration was longer than the recommended 7 h minimum per night [23] in TRE (08:10 ± 01:27 h:min), CR (07:41 ± 01:20 h:min), and control groups (07:50 ± 01:30 h:min), indicating adequate sleep duration in all groups. Sleep duration did not change significantly between groups by month 6 or month 12. Wake time and sleep onset latency also did not change significantly between groups by month 6 or month 12. As for bedtime, the TRE group went to bed later at 6 months, compared to the CR group, but this effect was no longer noted at month 12. The CR group went to bed later, compared to the control group, by the end of the study.

At baseline, the insomnia severity score was 8.2 ± 5.9 for the TRE group, indicating subclinical insomnia (ISI score 8–14). The insomnia severity score was 7.4 ± 5.6 for CR and 7.0 ± 5.0 for controls, indicating no clinical insomnia (ISI score 0–7). Insomnia severity scores did not change significantly between groups by month 6 or 12. Changes in body weight and body composition were not related to any sleep measure in any group at month 6 or month 12.

## 4. Discussion

This is the first study to compare the long-term (12-month) effects of TRE to CR on sleep outcomes in adults with obesity. Our findings show that TRE produced significant reductions in body weight (~5%) after 12 months, but no changes in sleep quality, duration, insomnia severity, or risk of obstructive sleep apnea, versus CR and controls.

Obstructive sleep apnea is highly prevalent in those with obesity, particularly when BMI exceeds 40 kg/m^2^ [24]. The mechanisms relating obesity to sleep apnea risk involve excess deposition of fat in the tissues surrounding the upper airway [25]. This can result in a smaller lumen and increased collapsibility of the upper airway, predisposing an individual to apnea [25]. In our study, 26–54% of subjects with obesity were at high risk of sleep apnea. After 12 months, no significant changes in the risk of sleep apnea were noted, even though our TRE and CR subjects lost 5% of body weight, versus controls. However, it is possible that our interventions did not achieve enough weight reduction to improve this sleep metric. Findings suggest that at least 10% weight loss may be necessary to decrease the risk of obstructive sleep apnea in people with obesity [26].

Sleep quality and duration (measured by PSQI) remained unchanged in all groups after 12 months. Our findings are consistent with the intermittent fasting sleep literature. For instance, Teong et al. [27] conducted a 2-month experiment comparing the effects of CR versus alternate day fasting (ADF; 500 kcal fast day alternated with an ad libitum feast day) on sleep quality using PSQI. The study found no changes in sleep quality in either the CR or ADF group after 2 months with 4–6% weight loss [27]. Similarly, Martin et al. [28] reported no changes in sleep quality, sleep latency, or sleep duration after two years of CR compared to controls, despite 12% weight loss. A recent systematic review examined the effect of TRE on sleep and included six TRE studies [12]. Almost all the studies included in the review [12] showed no change in sleep quality after 1–3 months of TRE, with 2–3% weight loss. It is possible that sleep quality and duration were not improved in any of these TRE trials because the degree of weight loss fell short of being clinically significant (>5% weight loss). Evidence suggests that weight loss may improve sleep quality and duration by reducing sleep fragmentation and alleviating sleep-disordered breathing, but higher amounts of weight loss (>5% from baseline) are most likely needed to observe these effects [29].

Insomnia severity was also assessed. At the beginning of the study, participants in the TRE group portrayed sub-clinical insomnia, while subjects in the CR and control groups displayed no clinically significant insomnia. By the end of the trial, no changes in insomnia severity were demonstrated in any group. This finding is not surprising, as our subjects did not portray clinically significant insomnia at the onset of treatment; therefore, it would be unlikely for this sleep parameter to improve. This result is complementary to other TRE studies, which also report no change in insomnia severity after 2–3 months of this dietary intervention [12].

Physical activity and mental health can also impact sleep outcomes and may have potentially confounded our study findings. Regular exercise has been shown to improve sleep quality and decrease sleep onset latency [30,31]. In the present trial, physical activity (steps/day) did not change over time or between groups [14]. However, we did not measure other parameters of physical activity such as time spent in standing, walking, or exercising. Therefore, there is a possibility that we failed to capture small changes in our participant’s physical activity that may have impacted sleep.

Another potential confounder is mental health. Certain mental health conditions, such as depression and anxiety, are correlated with reduced sleep quality and increased risk of insomnia [32,33]. We previously conducted a secondary analysis on self-reported mood, anxiety, and depression scores, and found no changes in these variables over 12 months in the TRE and CR groups, versus controls [34]. Thus, it is unlikely that these mental health factors confounded our findings, but they should still be taken into consideration when interpreting our sleep findings.

This trial is innovative in a couple ways. This is the first randomized controlled study to compare the effects of TRE versus standard care (daily CR) on sleep in people with obesity. Previous studies only investigated the independent effects of either TRE or CR, without a direct comparison between the two. Moreover, this is the first study to examine the long-term effects of TRE on sleep (i.e., over 12 months). All previous studies assessing TRE on sleep were quite short (i.e., 1–3 months). Thus, this trial is novel in that it documents how sleep changes in relation to weight loss by TRE over a full year.

Our study has several limitations. First, our sample size was small (*n* = 90), and our power calculation was based solely on changes in body weight. We performed a post hoc power analysis and found that the computed achieved power for sleep quality was 58%, for sleep duration was 29%, for sleep onset latency was 64%, and for insomnia severity was 21%. Thus, the lack of effect on sleep variables could very likely be due to small sample size. Second, all outcomes of sleep measures were self-reported. Future studies in this area should implement wrist actigraphy to provide more objective assessments of rest and activity patterns. Third, we did not quantify many of the confounding variables that can impact sleep such as stress, work/travel schedules, and timing of caffeine intake. These confounders should be accounted for in future studies in this area. Fourth, we did not exclude participants who were taking sleep-impacting medications or dietary supplements. Though only a few participants reported taking these agents (i.e., magnesium), this design flaw should be taken into consideration when interpreting the present findings.

## 5. Conclusions

In summary, the weight loss induced by 12 months of TRE (~5% from baseline) does not improve sleep quality, duration, insomnia severity, or risk of obstructive sleep apnea in individuals with obesity, relative to CR and controls. However, these findings will need to be confirmed by a well-powered randomized controlled trial specifically designed to assess the impact of TRE on sleep in this population group.

## Figures and Tables

**Table 1:** Baseline characteristics of the study participants.

	TRE	CR	CON	*p*-Value
***n***	30	30	30	
**Age (y)**	44 ± 12	44 ± 9	44 ± 13	-
**Sex, No. (%)**				-
Female	25 (83%)	24 (80%)	25 (83%)	-
Male	5 (17%)	6 (20%)	5 (17%)	-
**Race or ethnic group, No. (%)**				0.643
Black	11 (37%)	9 (30%)	10 (33%)	
Asian	3 (10%)	3 (10%)	0 (0%)	
Hispanic	13 (43%)	11 (37%)	17 (57%)	
White	3 (10%)	7 (23%)	3 (10%)	
**Body weight & composition**				
Body weight (kg)	100 ± 17	102 ± 18	102 ± 17	0.800
Fat mass (kg)	46 ± 11	47 ± 11	47 ± 10	0.833
Lean mass (kg)	50 ± 10	50 ± 9	51 ± 8	0.835
Visceral fat mass (kg)	1.6 ± 0.6	1.6 ± 0.8	1.7 ± 0.8	0.883
Waist circumference (cm)	109 ± 13	110 ± 14	110 ± 13	0.940
Height (cm)	164 ± 9	166 ± 9	165 ± 7	0.557
Body mass index (kg/m^2^)	37 ± 6	37 ± 5	38 ± 5	-
**Morningness–Eveningness Questionnaire (MEQ)**				
Total score	52 ± 9	56 ± 10	54 ± 8	0.259
**Berlin Questionnaire**				
High risk of obstructive sleep apnea (% occurrences)	54	38	26	0.094
**Pittsburgh Sleep Quality Index (PSQI)**				
Total sleep quality score	7.8 ± 3.8	6.8 ± 3.3	8.1 ± 2.4	0.336
Wake time (h:min)	06:37 ± 01:41	06:19 ± 1:15	06:27 ± 01:20	0.411
Bedtime (h:min)	22:27 ± 00:54	22:38 ± 0:51	22:37 ± 00:51	0.117
Sleep duration (h:min)	08:10 ± 01:27	07:41 ± 1:20	07:50 ± 01:30	0.463
Sleep latency score	1.3 ± 1.0	1.0 ± 1.0	1.6 ± 0.7	0.504
**Insomnia Severity Index (ISI)**				
Total score	8.2 ± 5.9	7.4 ± 5.6	7.0 ± 5.0	

Abbreviations: CON: control group, CR: calorie restriction group, ISI: Insomnia Severity Index, MEQ: Morningness–Eveningness Questionnaire, PSQI: Pittsburgh Sleep Quality Index, TRE: time-restricted eating group. Continuous variables reported as mean ± SD. Participants at high risk of obstructive sleep apnea reported as % occurrences. *p*-value: ANOVA for continuous variables; McNemar test for categorical variables.

**Table 2:** Change in body weight, body composition, sleep parameters by month 6 and 12.

Variables	Change from Baseline (95% CI)			Difference Between Groups (95% CI)		
	Time-Restricted Eating (TRE)	Daily Calorie Restriction (CR)	Control (CON)	TRE vs. CR	TRE vs. CON	CR vs. CON
**Body weight & composition**						
Body weight (kg)						
Month 6	−4.00 (−5.31, −2.70)	−5.14 (−7.66, −2.62)	0.00 (−1.40, 1.40)	1.14 (−1.63, 3.91)	**−4.00 (−5.87, −2.13) ^a^**	**−5.14 (−7.95, −2.33) ^a^**
Month 12	−3.49 (−5.65, −1.32)	−4.30 (−7.63, −0.96)	1.12 (−0.69, 2.94)	0.81 (−3.07, 4.69)	**−4.61 (−7.37, −1.85) ^a^**	**−5.42 (−9.13, −1.71) ^a^**
Body weight (%)						
Month 6	−4.27 (−5.69, −2.84)	−5.06 (−7.56, −2.57)	−0.03 (−1.47, 1.40)	0.80 (−2.01, 3.61)	**−4.23 (−6.21, −2.26) ^b^**	**−5.03 (−7.84, −2.22) ^b^**
Month 12	−3.76 (−5.89, −1.64)	−4.20 (−7.59, −0.80)	1.11 (−0.72, 2.94)	0.43 (−3.48, 4.34)	**−4.87 (−7.61, −2.13) ^b^**	**−5.30 (−9.06, −1.54) ^b^**
Fat mass (kg)						
Month 6	−2.68 (−3.75, −1.61)	−2.25 (−4.62, 0.13)	−0.13 (−1.33, 1.08)	−0.43 (−2.96, 2.10)	**−2.55 (−4.12, −0.98) ^b^**	−2.12 (−4.71, 0.46)
Month 12	−2.20 (−3.88, −0.52)	−2.61 (−5.97, 0.74)	0.57 (−1.14, 2.27)	0.42 (−3.24, 4.07)	**−2.77 (−5.10, −0.43) ^b^**	−3.18 (−6.85, 0.49)
Lean mass (kg)						
Month 6	−0.12 (−0.97, 0.72)	−0.23 (−1.07, 0.61)	0.23 (−0.26, 0.72)	0.11 (−1.05, 1.27)	−0.36 (−1.31, 0.60)	−0.46 (−1.41, 0.48)
Month 12	−0.41 (−0.91, 0.08)	−0.74 (−1.44, −0.03)	0.39 (−0.51, 1.29)	0.32 (−0.52, 1.16)	−0.81 (−1.81, 0.20)	**−1.13 (−2.24, −0.01) ^b^**
Visceral fat mass (kg)						
Month 6	−0.22 (−0.30, −0.14)	−0.19 (−0.34, −0.03)	−0.05 (−0.15, 0.05)	−0.03 (−0.20, 0.14)	**−0.17 (−0.29, −0.05) ^b^**	−0.14 (−0.32, 0.04)
Month 12	−0.14 (−0.23, −0.04)	−0.12 (−0.29, 0.06)	−0.03 (−0.16, 0.10)	−0.02 (−0.22, 0.17)	−0.11 (−0.27, 0.06)	−0.08 (−0.30, 0.13)
Waist circumference (cm)						
Month 6	−5.54 (−7.51, −3.57)	−4.69 (−7.75, −1.63)	−0.70 (−2.29, 0.88)	−0.85 (−4.40, 2.70)	**−4.83 (−7.30, −2.37) ^b^**	−3.98 (−7.35, −0.62)
Month 12	−6.44 (−8.65, −4.24)	−3.77 (−7.46, −0.08)	−1.46 (−3.77, 0.84)	−2.67 (−6.86, 1.52)	**−4.98 (−8.09, −1.87) ^b^**	−2.30 (−6.55, 1.94)
**Berlin Questionnaire**						
High risk of obstructive sleep apnea (% occurrences)						
Month 6	−4 (−25, 18)	−10 (−33, 13)	6 (−15, 27)	6 (−25, 37)	−9 (−39, 20)	−16 (−46, 15)
Month 12	−14 (−32, 4)	4 (−18, 26)	−3 (−25, 18)	−18 (−46, 10)	−11 (−38, 17)	7 (−23, 37)
**Pittsburgh Sleep Quality Index (PSQI)**						
Total sleep quality score						
Month 6	−1.24 (−2.86, 0.38)	−0.06 (−1.42, 1.31)	−1.23 (−2.25, −0.21)	−1.18 (−3.25, 0.88)	−0.01 (−1.88, 1.86)	1.17 (−0.49, 2.83)
Month 12	−1.08 (−2.40, 0.24)	−0.94 (−1.88, 0.01)	−1.26 (−2.43, −0.08)	−0.14 (−1.73, 1.44)	0.18 (−1.55, 1.90)	0.32 (−1.15, 1.79)
Wake time (h:min)						
Month 6	00:07 (−00:20, 00:34)	−00:01 (−00:42, 00:41)	00:08 (−00:21, 00:37)	00:07 (−00:41, 00:55)	−00:02 (−00:40, 00:37)	−00:08 (−00:58, 00:41)
Month 12	−00:13 (−00:48, 00:23)	00:04 (−00:29, 00:37)	00:04 (−00:24, 00:32)	−00:16 (−01:04, 0:31)	−00:16 (−01:00, 0:28)	00:00 (−00:42, 00:43)
Bedtime (h:min)						
Month 6	00:24 (00:02, 00:46)	−00:13 (−00:37, 00:12)	00:07 (−00:08, 00:23)	**00:37 (00:05, 01:08) ^b^**	00:17 (−00:10, 00:43)	−00:20 (−00:48, 00:08)
Month 12	−00:01 (−0:32, 00:31)	−00:26 (−00:53, 00:02)	00:08 (−00:10, 00:25)	00:25 (−00:16, 01:05)	−00:08 (−0:43, 0:26)	**−00:34 (−01:05, −00:02) ^b^**
Sleep duration (h:min)						
Month 6	−00:22 (−00:46, 00:02)	−00:01 (−00:40, 00:39)	−00:02 (−00:41, 00:37)	−00:22 (−01:07, 00:23)	−00:20 (−01:05, 00:25)	00:01 (−00:53, 00:56)
Month 12	−00:12 (−00:50, 00:26)	00:30 (−00:08, 01:08)	−00:04 (−00:37, 00:28)	−00:42 (−01:34, 00:10)	−00:08 (−00:56, 00:41)	00:34 (−00:14, 01:23)
Sleep latency score						
Month 6	−0.16 (−0.46, 0.15)	−0.21 (−0.58, 0.16)	−0.51 (−0.81, −0.21)	0.05 (−0.42, 0.52)	0.35 (−0.07, 0.77)	0.30 (−0.17, 0.76)
Month 12	−0.08 (−0.42, 0.27)	−0.17 (−0.49, 0.15)	−0.46 (−0.93, 0.01)	0.10 (−0.36, 0.55)	0.38 (−0.18, 0.95)	0.29 (−0.27, 0.84)
**Insomnia Severity Index (ISI)**						
Total score						
Month 6	−1.30 (−3.10, 0.50)	−0.94 (−3.23, 1.35)	−0.43 (−2.26, 1.40)	−0.36 (−3.20, 2.48)	−0.87 (−3.37, 1.63)	−0.51 (−3.37, 2.35)
Month 12	−1.09 (−2.69, 0.51)	−1.50 (−3.33, 0.33)	−1.12 (−2.92, 0.68)	0.41 (−1.96, 2.79)	0.04 (−2.31, 2.38)	−0.38 (−2.88, 2.13)

Abbreviations: CON: control group, CR: calorie restriction group, ISI: Insomnia Severity Index, PSQI: Pittsburgh Sleep Quality Index, TRE: time-restricted eating group. Means were estimated using an intention-to-treat analysis using a linear mixed model with 95% CIs for each parameter from baseline by diet group. ^a^ Indicates statistical significance using Bonferroni-adjusted 2-tailed *p* < 0.017. ^b^ Indicates statistical significance using *p* < 0.05. Bold indicates significant effects.

## Data Availability

The original contributions presented in the study are included in the article, further inquiries can be directed to the corresponding authors.

## Trial Registration

Clinicaltrials.gov, NCT04692532.
